# Health Tract, No. 292

**Published:** 1867-05

**Authors:** 


HEALTH TRACT, No. 292.

### LIFE'S BATTLE.

"When you've got a big thing to do, go ahead," were words of wisdom uttered in our hearing by a youth of fourteen, to little "Robbie" as he sat and contemplated discouragingly, a task which had been assigned him, which, in his youthful imagination seemed to be an interminable job. Many a reader will call to remembrance, times when he seemed to have so much to do, that he did not know where to begin, and many a valuable hour has been wasted in trying to come to a conclusion what to do first, under such circumstances there can be no better plan to carry out the suggestion of Augustus C. "When you've got a big thing to do, go ahead." He was a tall boy; fresh from the country, and possessing exhuberant health, and a frank, cheerful, generous nature, with his heart in his hand, he was ever ready to help a friend, and would do it with a will. Feeble minds magnify difficulties and perform enough labor to do half the work, in merely clearing away the rubbish. Those dilatory folk who can never "make up their minds; who halt and hesitate and "back and fill" so painfully, that existence is a burden, and half the time, when they do come to a conclusion they change to the opposite from very fear that they have made an unwise choice. Such persons lead a life of inefficiency, of almost worthlessness, and when they die, "are not missed." If the reader has such an unfortunate and really painful, mental malady, he may derive some consolation from the reflection that it is not wholly irremediable, because it is a quality largely depending on the bodily condition and the mental habits. Vigorous, manly health makes one feel as if he could sweep away every obstacle before him with a wave of the hand; that health should be striven for in an active life, which keeps the body "on the go;" which taxes the mental energies to an extent that compels them to be busy, not over crowded but always a little pushed; an additional element of success would be to secure such a person an occupation involving responsibilities; involving the direction of others; a situation which implies confidence reposed by persons of character and position, in this way work, business, becomes a pleasure, and mind and body being fully occupied, the capabilities of both will be invited out, will be cherished, strengthened, and grow to fair proportions from very use. Wake up then ye hesitating, halting ones, and make a manful fight against whatever of obstacles may stand in the way of success in life; stand up to them face to face; sometimes they will melt away as snow before the silent warming sun, at others they may be too numerous to mention, but when they seem to be, take courage and they will oftentimes vanish like the morning dew, in the practice of "Gussies'" plan, "When you've got a big thing to do, go ahead."

